# Microplastic Exposure Disrupts Energy Homeostasis and Welfare in Goldfish

**DOI:** 10.3390/ani16091381

**Published:** 2026-04-30

**Authors:** Lisbeth Herrera-Castillo, Nerea Navajas-Jiménez, André Barany, Esther Isorna, Miguel Gómez-Boronat, Nuria de Pedro

**Affiliations:** Department of Genetics, Physiology and Microbiology, Faculty of Biological Sciences, Complutense University of Madrid, 28040 Madrid, Spain; lisbethh@ucm.es (L.H.-C.); nernav01@ucm.es (N.N.-J.); abarany@ucm.es (A.B.); eisornaa@ucm.es (E.I.); miguelgomezboronat@ucm.es (M.G.-B.)

**Keywords:** polystyrene microspheres, energy balance, feeding, body weight, anxiety-like behavior, metabolic rate, stress, circadian rhythms, *Carassius auratus*

## Abstract

The accumulation of microplastics in aquatic ecosystems poses a significant threat to fish physiology and welfare. This study investigated the impact of exposure to virgin polystyrene microplastics (15 µm) on energy balance and welfare in goldfish (*Carassius auratus*). Fish were exposed for 14 days, and the effects were assessed through an integrated analysis of behavioral, metabolic, neuroendocrine, and physiological parameters. Microplastic exposure significantly reduces feed intake and feed anticipatory activity, indicating a potent anorexigenic effect. This effect was driven by neuroendocrine disruption, characterized by the downregulation of orexigenic neuropeptides (*npy*, *agrp*, *hcrt*) and the upregulation of anorexigenic signaling (*pomca*, *cartpt*, *lepa*). Simultaneously, exposed fish exhibited increased oxygen consumption, suggesting elevated metabolic demands. These factors converged to impaired growth and reduced hepatosomatic index, suggesting altered energy allocation. Furthermore, microplastic exposure induced anxiety-like responses and increased plasma cortisol levels, confirming the activation of the physiological stress response. Overall, these findings demonstrate that microplastics disrupt energy homeostasis and trigger behavioral shifts that ultimately compromise fish welfare and the biological resilience of aquatic species.

## Simple Summary

While microplastics are proliferating in freshwater environments, their impact on fish nutrition and welfare is not yet fully understood. This study examined how a 14-day exposure to microplastics affects feeding, energy expenditure, stress and anxiety-like behavior in goldfish. Our results show that fish exposed to microplastics suppressed appetite, diminished feed anticipation, and increased energy expenditure, leading to stunted growth and the depletion of hepatic energy reserves. Furthermore, exposed fish also displayed signs of stress and anxiety, compromising overall well-being. Together, these findings show that microplastics disrupt energy balance and welfare in fish, suggesting that their impact extends beyond mere survival. These results highlight the importance of considering integrated behavioral and physiological indicators when assessing their effects in fish.

## 1. Introduction

Global reliance on plastics has escalated at an unprecedented rate, with annual production already surpassing hundreds of millions of tons and projections indicating further dramatic increases [1,2]. The widespread use of disposable plastic items combined with inadequate waste management has resulted in the continuous leakage of plastic debris into aquatic ecosystems [3,4]. Over time, larger plastic materials fragment into smaller particles known as microplastics (1 μm to 5 mm), which are now recognized as one of the most pervasive pollutants in aquatic environments [2,5,6]. Research has predominantly focused on marine environments, leaving a significant knowledge gap regarding freshwater ecosystems [7,8], despite growing evidence of considerable microplastic contamination in freshwater aquaculture systems and freshwater fish, including cyprinid species, reported in recent studies [9,10,11].

Microplastics are highly persistent and mobile, interacting with a wide range of aquatic organisms and transferring across trophic levels. They have been detected in diverse aquatic species, including zooplankton, mollusks, crustaceans, and fish [1,12,13]. Moreover, the detection of microplastics in commercially important species underscores their relevance not only for aquatic ecosystems but also for food safety and potential human exposure [14,15,16]. In fish, once ingested, microplastics initially accumulate in the gastrointestinal tract, where they can interfere with digestion and nutrient assimilation, while additional exposure routes such as the gills may allow particle adhesion or penetration into respiratory tissues [17,18]. From these entry routes, microplastics can translocate to internal organs such as the liver, muscle, and brain, raising concerns about systemic toxicity [8]. Microplastic exposure to fish has been associated with a broad spectrum of adverse effects affecting multiple levels of biological organization. Experimental studies have shown that microplastics induce cellular and tissue damage, oxidative stress, and inflammatory responses, as well as physiological alterations affecting several systems, including digestive, neuroendocrine, and reproductive systems [8,19,20,21]. Beyond these physiological effects, microplastics can also alter behavior in fish, including feeding, locomotion, predator avoidance, and social interactions [21,22,23,24,25,26]. Such behavioral changes are progressively recognized as sensitive indicators of organismal fitness, often revealing sublethal effects that may precede overt toxicity, and complementing conventional toxicological analyses. Despite the increasing evidence of microplastic toxicity, significant gaps remain regarding their sublethal effects on energy homeostasis and welfare. This is particularly true for freshwater species such as the goldfish (*Carassius auratus*), a species belonging to the Cyprinidae, a family that dominates global freshwater aquaculture production [27,28], making it an ecologically and economically relevant model for assessing plastic pollution. Previous research in goldfish has addressed microplastic ingestion and egestion dynamics [29], ionoregulatory disturbances at the gills [30], effects on oxidative stress, liver damage [31,32] and impaired feeding by disrupting feeding regulators [33]. However, these studies generally focus on isolated endpoints rather than adopting an integrative perspective. The present study addresses this gap by combining these endpoints to provide a more comprehensive understanding of how microplastics affect energy homeostasis and welfare.

Energy homeostasis, defined by the balance between intake (feeding behavior) and expenditure (metabolism and activity), is fundamental for growth, reproduction, and survival, and its disruption may trigger cascading consequences that extend beyond individual physiology to population-level processes. Feed intake in fish is regulated by an integrated hypothalamic neuroendocrine system, in which orexigenic neurons expressing *neuropeptide Y* (NPY) and *agouti-related peptide* (AgRP) stimulate feeding, whereas anorexigenic neurons expressing *proopiomelanocortin* (POMC) and *cocaine- and amphetamine-regulated transcript* (CART) inhibit it [34,35,36]. This hypothalamic circuit integrates peripheral hormonal signals, primarily leptin and ghrelin, which exert opposing actions: leptin (mainly hepatic in teleosts) promotes satiety by activating POMC/CART neurons and inhibiting NPY/AgRP neurons [35,36,37], whereas ghrelin (produced in the gastrointestinal tract) stimulates feed intake through the opposite effects [34]. This gut–liver–brain axis ensures fine-tuned regulation of energy balance but may be particularly sensitive to environmental stressors such as microplastics. Although several studies have evaluated growth performance after microplastic exposure, few studies have examined direct effects on feed intake, and only a limited number have addressed the underlying neuroendocrine regulatory mechanisms. Reported effects on appetite-related signals remain inconsistent across species and particle types [33,38], highlighting the need for integrative approaches combining behavioral and molecular endpoints to better understand how microplastics disrupt energy homeostasis.

In fish, physiological stress responses are closely linked to the regulation of energy balance and welfare and are primarily mediated by activation of the hypothalamus–pituitary–interrenal (HPI) axis, leading to the release of cortisol, which promotes energy mobilization and adaptive responses to environmental challenges [39,40,41]. Under stress conditions, sustained elevation of cortisol levels is commonly observed, making this hormone a widely accepted biomarker of fish welfare [41,42,43]. Environmental stressors can also induce anxiety-like behavioral responses in fish, including altered risk assessment and reduced exploratory behavior, which are indicative of compromised welfare [44,45,46]. These behavioral alterations can be reliably assessed using non-invasive approaches, such as open field and black–white preference tests, which are widely applied in fish welfare research [47,48,49]. Together, endocrine and behavioral responses provide complementary indicators of fish welfare and allow the assessment of how environmental stressors such as microplastics may disrupt both physiological stability and adaptive behavior.

This study aims to determine the impact of microplastic exposure on energy homeostasis and welfare in fish through an integrated analysis of behavioral, physiological, neuroendocrine, and metabolic responses. The model species selected was the goldfish (*Carassius auratus*) for its well-established use in neuroendocrine and toxicological research [50] and its ecological relevance as a representative freshwater cyprinid. Using this teleost freshwater model, we focused on two complementary axes. First, feed intake, feed anticipatory activity, metabolic rate, and locomotor activity were evaluated as key indicators of the balance between energy intake and expenditure, and their disruption was examined in relation to growth performance. Second, anxiety-like behavior and circulating cortisol levels were assessed as relevant biomarkers of organism welfare. Furthermore, we investigated the neuroendocrine mechanisms underlying these responses to elucidate how microplastics disrupt physiology and behavior linked to molecular mechanisms.

## 2. Materials and Methods

### 2.1. Fish and Housing

Goldfish were distributed into four tanks (*n* = 6 fish per tank), with 60 L of aerated and filtered fresh water. Initial body weight (BW) was 16.54 ± 2.27 g in the control group and 16.32 ± 2.48 g in the microplastic-exposed group (mean ± Standard Deviation). Fish were kept in controlled temperature conditions (21 ± 1 °C), with a photoperiod of 12 h of light (8:00–20:00) and 12 h of darkness (20:00–8:00). Daily feed intake took place at 10:00 h with commercial dry pellets (Sera Pond Granulat; Heinsberg, Germany) with a ration equivalent to 1.5% BW. Fish were acclimated to these conditions at least three weeks prior to experimentation.

### 2.2. Experimental Design

This work was carried out in accordance with current regulations (RD 53/2013, 2010/63/EU), and all experimental procedures were approved by the Animal Experimentation Committee of the Complutense University of Madrid and the Community of Madrid (PROEX 317.7/23).

The fish were divided into two experimental groups (*n* = 12 fish per group), with each group conducted in duplicate tanks (6 fish per tank). One group was the control group (C), with no exposure to microplastics, while the other group (MP) was exposed for 14 days to additive-free polystyrene microspheres (15 µm diameter, #74964, Sigma Aldrich, Madrid, Spain) at a concentration of 0.5 mg/L. The 14-day exposure period was selected as a short but sufficient timeframe to detect changes in energy homeostasis and growth-related responses. This concentration was selected based on previous experimental studies in fish [32,33,38,51], and lies within the range of microplastic concentrations considered environmentally relevant in aquatic systems [52,53,54]. Continuous aeration was applied to ensure homogeneous dispersion of microplastic particles in the water and to maintain particles in suspension throughout the exposure period. The stability of the suspension was periodically checked by light microscopic observation, which did not indicate marked aggregation under the experimental conditions used. To maintain appropriate hygienic conditions while preventing microplastic loss, the filtration system was deliberately omitted, and 50% of the water volume was renewed daily. Although daily water renewal may introduce minor handling effects, these were applied equally across treatments and are therefore unlikely to have influenced the differences observed between experimental groups. Following each water exchange, microplastics were added every day at 12:00 to replenish the concentration and maintain consistent exposure levels throughout the experiment.

Before the experiment, fish were anesthetized using tricaine methanesulfonate (MS-222, 0.16 g/L, #E10521, Sigma Aldrich, Madrid, Spain), buffered with 288 mg of sodium bicarbonate (#BCCB0204, Sigma Aldrich, Madrid, Spain) to achieve a pH of 7.2, and were marked for identification via subcutaneous injection of tattoo ink (Eternal Ink, Brighton, MI, USA) using 1 mL syringes with 0.3 mm diameter needles. Throughout the 14-day experimental period, daily data were obtained on body weight, standard length, feed intake, and locomotor activity. After 7 days of treatment (on day 8), the anxiety-like behavior of the fish was assessed using the black–white preference and open field tests. On day 14, individual feeding trials were conducted, and on day 15, respirometry assays were performed to determine metabolic rate. Individual feeding and behavioral and respirometry tests were conducted under 24 h fasting conditions to eliminate potential acute effects of recent feeding and to standardize metabolic measurements, avoiding confounding effects associated with specific dynamic action. On day 16, fish fasted for 48 h were anesthetized, and blood was collected from the caudal vein, centrifuged (5 min, 12,000 *g*, 4 °C), and plasma was stored at −20 °C for cortisol analysis. All groups were subjected to identical handling and sampling procedures to minimize potential stress-induced variation associated with blood collection. Fish were then euthanized with an overdose of MS-222 (0.4 g/L), and the hypothalamus, anterior intestine, perivisceral fat, and liver were extracted. The latter two were weighed to calculate organosomatic indices. Hypothalamus, anterior intestine and liver samples were stored at −80 °C for subsequent gene expression analysis of different feeding regulators and transcription factors.

### 2.3. Physiological, Behavioral and Molecular Analysis

#### 2.3.1. Feed Intake

Daily feed intake was monitored between 10:00 and 12:00 in each tank (six fish per tank), except on day 1 (before the first microplastic exposure), day 8 (behavioral testing day), and day 14 (individual feeding trial). On the final day of treatment (day 14), intake was measured individually in 5 L tanks, following the same time schedule, as previously described [55].

Feed Intake (mg/g BW)=((Wi−Wf)×F)/BW

*Wi*, initial feed weight; *Wf*, final feed weight; *F*, dilution factor (1.15), accounting for pellet dissolution in water, calculated experimentally using the same pellet type after 2 h immersion.

#### 2.3.2. Gene Expression Analysis

Relative transcript levels of target genes (i.e., feeding and metabolic regulators, Table 1), including *agrp*, *npy*, *cartpt1*, *cartpt2*, *hcrt*, and *pomca* (hypothalamus), *ghrelin* (anterior intestine), and *lepa1*, *ppara*, and *pparg* (liver), were quantified using real-time quantitative polymerase chain reaction (RT-qPCR). Total RNA (tRNA) was extracted from intestine, liver and hypothalamus samples using TRI Reagent (#T9424, Sigma Aldrich, Madrid, Spain) following mechanical homogenization (Ultra-Turrax T8, IKA, Staufen, Germany). RNA concentration and purity were assessed via spectrophotometry (NanoDrop 2000, Thermo Scientific, Waltham, MA, USA). Residual genomic DNA was removed using RQ1 RNase-Free DNase (#M6101, Promega, Madison, WI, USA), and tRNA was resuspended in DEPC water. cDNA synthesis was performed with 0.5 μg of tRNA using an RNase inhibitor (RNasin; #N215B, Promega, Madison, WI, USA), the SuperScript IV Reverse Transcriptase (#18090010, Invitrogen, Carlsbad, CA, USA) and random primers (#48190–011, Invitrogen, Carlsbad, CA, USA), following the manufacturer’s instructions. The RT-qPCR was conducted on 96-well plates. Each well contained 0.5 μL of reverse primer (10 μM), 0.5 μL of forward primer (10 μM), 1 μL of cDNA, and 5 μL of the SYBR Green Supermix (#1725124, Bio-Rad Laboratories, Hercules, CA, USA), with ultrapure water added to a final volume of 10 μL. Each sample was measured in duplicates; negative controls and a series of 1/3 serial dilutions of pooled cDNA were included to generate the standard amplification curve and verify reaction efficiency. All curves showed efficiencies between 95 and 110%, with r^2^ > 0.98. The PCR protocol consisted of initial denaturation (30 s at 95 °C) and 40 cycles (5 s at 95 °C, 30 s at 60 °C). A melting curve (65–95 °C, 0.5 °C/5 s increments) was made at the end to confirm the specificity of each specific couple of primers (Sigma Aldrich, Madrid, Spain).

Primer sequences for the target and reference gene (*actb*) are listed in Table 1. Relative expression was calculated using the 2^−ΔΔCt^ method [56], by normalizing first to the reference gene and then to the control group.

#### 2.3.3. Locomotor Activity

Locomotor activity was monitored during the last 6 days of the acclimation phase and the 14-day experimental period using 6 infrared sensors (E3S-AD12, Omron Corporation, Kyoto, Japan) affixed to the tank walls [57]. Two sensors were positioned beneath the feeders to detect feeding-related movements and record feed anticipatory activity (FAA) during the 2 h prior to feed administration, while the remaining four sensors were distributed across the mid and lower zones of the tank to assess general activity. Each sensor emitted a continuous infrared beam that was disrupted by fish movement, generating pulses recorded every 10 min via an actimeter linked to Adq16 software (Micronec, Madrid, Spain). Data were processed using El Temps^®^ (Prof. Antoni Díez Noguera, University of Barcelona, Barcelona, Spain) to generate actograms and daily activity profiles. Locomotor data were excluded on days 1, 8, and 14 due to handling, behavioral testing, and feed intake trials, totaling 11 recording days.

#### 2.3.4. Metabolic Rate

Oxygen consumption (MO_2_) was measured as an indicator of metabolic rate using an intermittent-flow respirometry system (Loligo Systems, Viborg, Denmark), following established protocols [58]. The system included four methacrylate chambers immersed in a temperature-controlled (21 ± 1 °C), aerated freshwater container, equipped with wash and recirculation pumps. Fish were individually placed in the chambers for a 24 h recording period. Oxygen levels and temperature were monitored using optode sensors connected to a Witrox-4 module, with data acquisition managed using AutoResp™ software (v2.3.0, Loligo Systems, Viborg, Denmark).

#### 2.3.5. Growth Performance

To determine growth performance during the experimental period, several biometric indices were calculated:

- Body Weight Gain $(\%)=[\left(BWf-BWi\right)/BWi]\times 100$
- Body Length Gain $\left(\%\right)=[\left(Lf-Li\right)/Li]\times 100$
- Specific Growth Rate $\left(\%/day\right)=[(LnBWf-LnBWi)/days\;of\;treatment]\times 100$
- Fulton’s condition factor $(K)=(BWf/{Lf}^{3})\times 100$
- Organosomatic Indices (%)=(OW/BWf)×100

*BWi*, initial body weight; *BWf*, final body weight; *Li*, initial standard length; *Lf*, final standard length; *OW*, organ (liver or perivisceral fat) weight.

#### 2.3.6. Behavioral Tests

Anxiety-like responses were evaluated through consecutive open field and black–white preference tests after 7 days of microplastic exposure as outlined in prior work [47]. Individual fish were transferred to a 5 L tank and allowed a 5 min habituation period prior to each assay. For the open field test, fish were released near the wall of a circular tank (50 cm diameter, 10 cm water depth), with the central 75% defined as the open zone and the outer 25% as the wall. For the black–white preference test, fish were released in the black side of a rectangular tank (47 cm length × 10 cm width, 10 cm water depth) divided into black and white halves (Maze Engineers, Skokie, IL, USA). Behavior was recorded for 10 min in each test and analyzed using EthoVision XT 17.5 software (Noldus, Wageningen, The Netherlands). The quantified behavioral parameters included the percentage of time spent in each predefined zone. Specifically, the proportion of time spent in the perimeter zone was used as the thigmotaxis index, while the percentage of time spent in the dark compartment of the black–white test was used as the scototaxis index. In addition to these zone-based indices, we also quantified the number of entries into each zone, the latency to the first entry, and the total distance traveled. Heat maps illustrating the mean of fish trajectories and zone occupancy for each experimental group were generated using the same software, based on a color scale. All analyses were performed automatically by the software without manual scoring, ensuring that data extraction was fully blind to treatment group.

#### 2.3.7. Plasma Cortisol Levels

Cortisol levels were quantified using an ELISA kit (Demeditec Diagnostics GmbH, Kiel, Germany), following the manufacturer’s protocol, previously validated for goldfish plasma [59,60]. Samples and standards (10 μL) were added in duplicate, with a six-point calibration curve ranging from 0 to 800 ng/mL. After incubation with 200 μL of enzyme conjugate (1 h, room temperature), wells were washed and incubated with tetramethylbenzidine substrate (30 min, in dark conditions). The reaction was stopped with 2 N HCl, and absorbance was measured at 450 nm using a SPECTROstar nano spectrophotometer (BMG Labtech, Ortenberg, Germany) operated with MARS V5. 02 R3 data analysis software for Microsoft Windows. Cortisol concentrations were interpolated from the standard curve.

### 2.4. Statistical Analysis

All statistical procedures and graphical representations were performed using Sigmaplot software 12.0 (Systat Software Inc., Illinois, CA, USA). Results are expressed as mean + standard error of mean (SEM). Prior to analysis, datasets were tested for normal distribution and homogeneity of variances using the Shapiro–Wilk and Levene tests, respectively. When assumptions were not met, data were transformed using logarithmic or square root functions. Comparisons between control and microplastic groups were performed using Student’s *t*-test. For locomotor activity and oxygen consumption plotted in bar charts + SEM, a two-way ANOVA was applied to evaluate the effects of treatment group and time of day. No significant interactions between factors were detected; therefore, only main effects were considered, and no post hoc comparisons were required. A one-way ANOVA was used to compare feed intake among fish tanks; when significant differences were found (*p* < 0.05), an SNK *post hoc* test was performed. Circadian rhythm parameters for locomotor activity were analyzed using the El Temps^®^ software v.313. For locomotor activity, rhythm acrophases were determined using Cosinor analysis, and their significance was assessed with the Rayleigh test. Rhythm periods were estimated using Sokolove–Bushell periodograms. All significance thresholds were set at *p* < 0.05. Oxygen consumption rhythms were analyzed using Cosinor Online, fitting data to a cosine function defined by the equation: Y = M + A × cos(πt/12 − ɸ), where *M* is the mesor (mean rhythm level), *A* the amplitude (peak deviation from mesor), *t* the time, and ɸ the acrophase (time of the peak value). Significance of rhythmicity was assessed via zero-amplitude testing [61].

## 3. Results

### 3.1. Feed Intake

**Figure 1:**
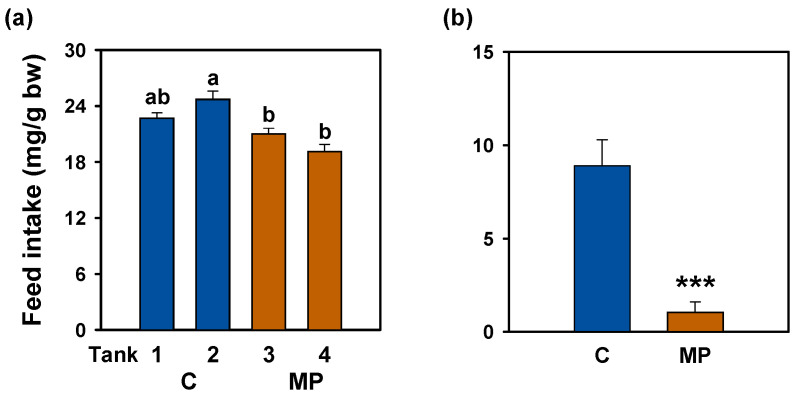
Feed intake after microplastic exposure in *Carassius auratus*. (**a**) Mean feed intake across 11 days for each experimental tank (*n* = 2 tanks per group). One-way ANOVA, *post hoc* Student–Newman–Keuls test, different letters indicate significant differences. (**b**) Individual feed intake on the last day of treatment (*n* = 12 fish/group). Student’s *t*-test, *** *p* < 0.001 compared to the control group. Data are presented as mean + SEM. C: control (blue); MP: microplastics (orange).

The exposure to microplastics over a 14-day period led to a pronounced decline in daily feed intake in goldfish. This pattern was consistent across tank-level measurements (six fish per tank; Figure 1a) and was further confirmed in individual assessments on the final day (Figure 1b), where intake showed an 88.3% decrease. Importantly, this divergence emerged early in the exposure period, as feed intake was already significantly reduced after 7 days (C = 24.21 ± 0.81 vs. MP = 20.30 ± 0.69; *p* = 0.0013). To rule out handling effects, feed intake was normalized to pre-exposure baseline values (the previous day of the treatment = 100%). At day 7, control tanks showed a positive increase to baseline (+18.7%), whereas microplastic-exposed tanks exhibited a marked reduction (−17%).

### 3.2. Feed Intake Regulators

**Figure 2:**
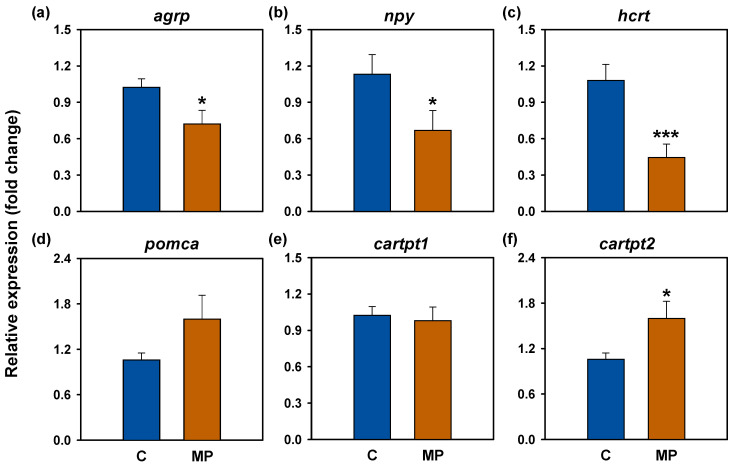
mRNA abundance of hypothalamic feeding regulators after microplastic exposure in *Carassius auratus*. Relative expression of orexigenic neuropeptides (**a**–**c**) and anorexigenic neuropeptides: (**d**–**f**) Relative expression values are normalized to the reference gene *actb*. Data are presented as mean + SEM (*n* = 10–12/group). Student’s *t*-test, * *p* < 0.05, *** *p* < 0.001 compared to the control group. C: control (blue); MP: microplastics (orange).

In the hypothalamus, microplastic exposure led to a significant downregulation of orexigenic genes, including *agrp*, *npy*, and *hcrt*, with *hcrt* showing the most pronounced decrease (Figure 2a–c). Conversely, among anorexigenic genes, *cartpt2* expression was significantly elevated (Figure 2f), while *pomca* exhibited a trend to increase (*p* = 0.058; Figure 2d). No changes were observed in *cartpt1* expression (Figure 2e).

**Figure 3:**
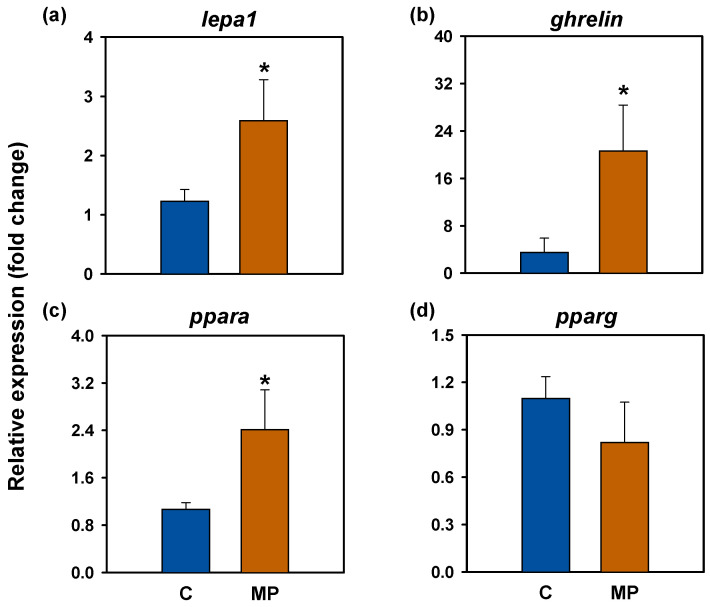
mRNA abundance of feeding regulators (**a**,**b**) and transcription factors (**c**,**d**) after microplastic exposure in *Carassius auratus*. Relative expression values are normalized to the reference gene *actb*. Data are presented as mean + SEM (*n* = 10–12/group). Student’s *t*-test, * *p* < 0.05 compared to the control group. C: control (blue); MP: microplastics (orange).

Hepatic expression of *lepa1* and *ppara* was significantly augmented after the microplastic exposure (Figure 3a,c), whereas *pparg* levels remained unchanged compared to controls (Figure 3d). Microplastics also induced upregulation of intestinal *ghrelin* (Figure 3b).

### 3.3. Locomotor Activity

**Figure 4:**
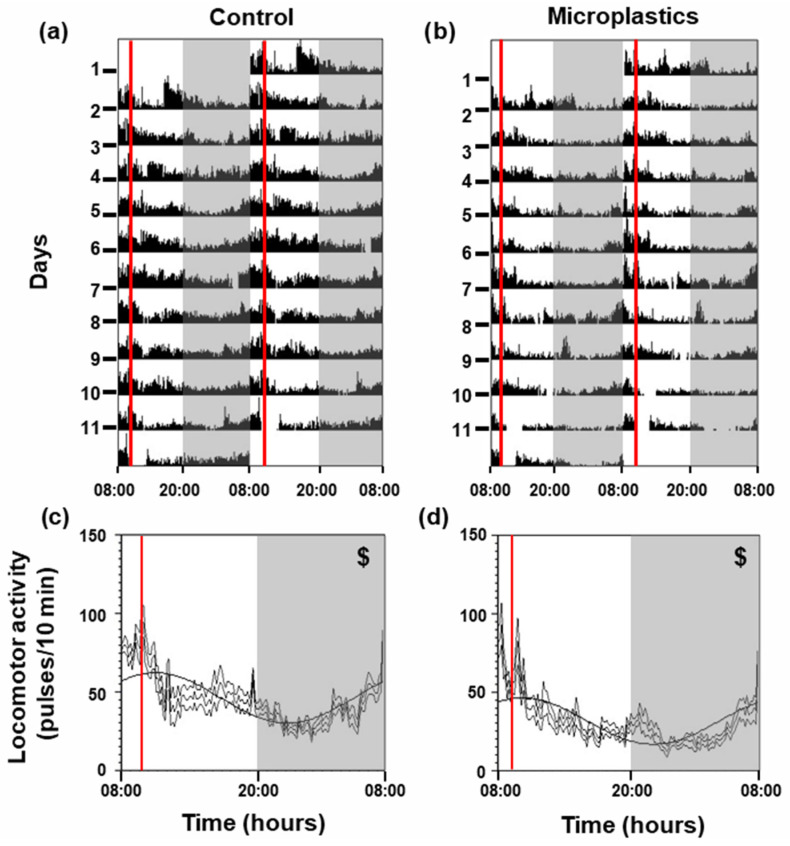
Locomotor activity of *Carassius auratus* exposed to microplastics for 14 days. (**a**,**b**) Representative actograms are illustrated in a double plot format (48 h time scale) for better visualization. (**c**,**d**) Average waveform of locomotor activity (mean ± SEM, *n* = 11 days), with periodic sinusoidal function wave represented by bold black line. Light and dark phases are indicated by white and gray areas, respectively. The red line shows the feeding time (10:00). Significance of the Rayleigh test for a 24 h rhythm is shown ($ *p* < 0.001).

**Figure 5:**
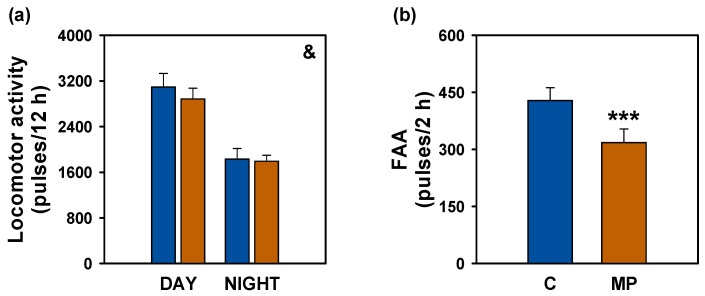
Locomotor activity of *Carassius auratus* after microplastic exposure. (**a**) General locomotor activity during day (08:00–20:00) and night (20:00–08:00). Two-way ANOVA showed a significant effect of time of day, & *p* < 0.001 day vs. night within each group. (**b**) Feed anticipatory activity (FAA, 08:00–10:00). Student’s *t*-test, *** *p* < 0.001 compared to the control group. Data are presented as mean + SEM (*n* = 11 days). C: control (blue); MP: microplastics (orange).

Actogram analyses revealed that both experimental groups exhibited elevated locomotor activity during the light phase (photophase, 08:00–20:00) compared to the dark phase (scotophase, 20:00–08:00), with an increase in movement observed in the hours preceding scheduled feeding (10:00) (Figure 4a,b). These activity patterns closely reflected those recorded during the six days prior to treatment initiation (Figure S1) and were further supported by the presence of statistically significant 24 h locomotor rhythms (*p* < 0.05) in both groups, observed before (Figure S1) and throughout the treatment period (Figure 4c,d). The control group showed a mesor of 46.04 pulses/10 min, whereas the microplastic group reached 31.53 pulses/10 min. Rhythm amplitude was 14.68 pulses/10 min in the control fish and 15.93 pulses/10 min in those exposed to microplastics. The acrophase, corresponding to the peak of locomotor activity, occurred at 11:06 in the control group and at 10:16 in the microplastics-exposed group. Notably, microplastic exposure did not alter overall activity levels during either the photophase or scotophase (Figure 5a), although FAA was markedly reduced in the microplastics-exposed group (*p* < 0.001; Figure 5b).

### 3.4. Metabolic Rate

**Figure 6:**
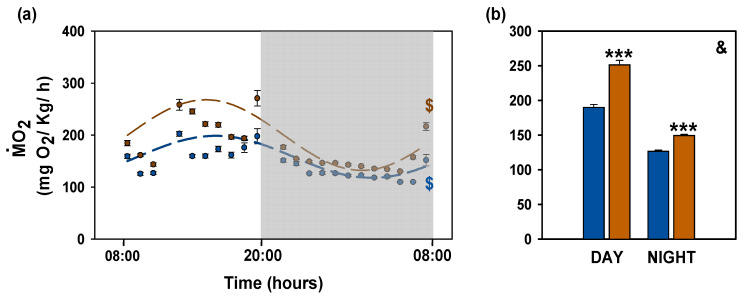
Metabolic rate of *Carassius auratus* after microplastic exposure. (**a**) Cosinor analysis of data of oxygen consumption (MO_2_) during a 24 h period, with data grouped by hour and presented as mean ± SEM. Blue and orange points represent control and microplastic groups, respectively. The fitted sinusoidal periodic functions are shown as dashed lines (blue, control; orange, microplastics). Zero amplitude test: $ *p* < 0.001. The shaded area indicates the dark phase (20:00–08:00). (**b**) Oxygen consumption during day and night. Two-way ANOVA test revealed significant effects of treatment (***) and time of day (&): *** *p* < 0.001, differences between control and microplastics; & *p* < 0.001, differences between day and night. Data are presented as mean + SEM (*n* = 8/group). Control (blue); microplastics (orange).

A robust circadian rhythm in oxygen consumption was detected in both control and microplastics-exposed goldfish (Figure 6a). Cosinor analysis revealed a mesor of 158.27 mg O_2_·kg^−1^·h^−1^ in the control group, whereas the microplastics-exposed fish showed a higher mesor (200.14 mg O_2_·kg^−1^·h^−1^). Rhythm amplitude was also increased in the microplastic group (67.91 mg O_2_·kg^−1^·h^−1^) compared to the control (40.43 mg O_2_·kg^−1^·h^−1^). Acrophase remained consistent between groups (15:47 h for control vs. 15:02 h for microplastics). Consistent with these results, exposure to microplastics over 14 days significantly elevated oxygen consumption during both diurnal and nocturnal periods (*p* < 0.001; Figure 6b).

### 3.5. Growth and Biometric Indices

Fish exposed to microplastics exhibited a significant reduction in body weight and length gain and the standard growth rate (Table 2). In the case of organosomatic indices, a significant decrease in the hepatosomatic index (HSI) was observed in the microplastic group (Table 2). No statistically significant differences were detected in the perivisceral fat index (PFI), although a trend toward lower values was observed in the microplastic group. No mortality was recorded in either the control or the microplastic treatment group during the experimental period.

### 3.6. Anxiety-like Behavior

**Figure 7:**
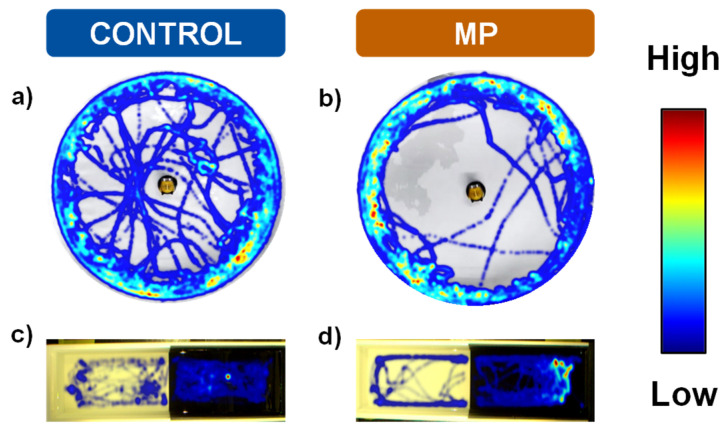
Heatmaps and swimming trajectories of *Carassius auratus* from control and microplastics groups in the open field ((**a**,**b**): mean of 12 fish/group) and the black–white preference tests ((**c**,**d**): black side at right; mean of 6 fish/group) generated using EthoVision XT 17.5 software (Noldus). The time spent in the different areas of the arena is represented by a color gradient ranging from cool colors (blue-green, shorter time spent) to warm colors (yellow-red, longer time spent).

Figure 7 illustrates the heat maps and movement trajectories obtained during the open field (Figure 7a,b) and black–white preference (Figure 7c,d) tests after 7 days of microplastic exposure. Fish exposed to microplastics exhibited clear avoidance of aversive zones in both paradigms (open zone and white compartment, respectively).

**Figure 8:**
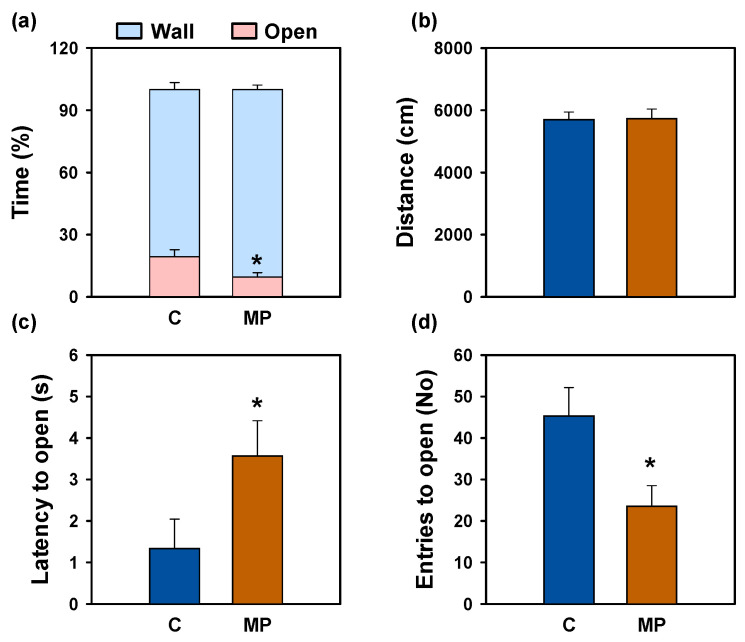
Behavioral parameters of the open field test after microplastic exposure in *Carassius auratus*. (**a**) Thigmotaxis index (% time in wall and open zones), (**b**) total distance travelled, (**c**) latency to enter the open zone, and (**d**) number of entries into the open zone. Data are presented as mean + SEM (*n* = 12/group). Student’s *t*-test, * *p* < 0.05 compared to the control group. C: control (blue); MP: microplastics (orange).

In the open field test, behavioral response observed in heat maps was supported by quantitative analyses, as fish exposed to microplastics spent significantly more time near the walls, which indicates a higher thigmotaxis index (Figure 8a), showed a longer latency to first entry (Figure 8c) and entered the open zone less frequently (Figure 8d). No significant differences were detected between groups in the total distance traveled (Figure 8b).

**Figure 9:**
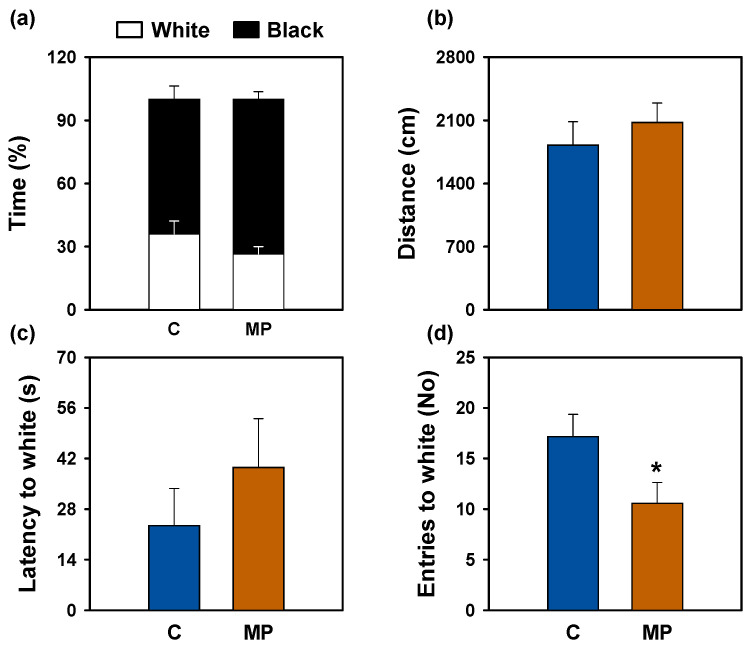
Behavioral parameters of the black–white preference test after microplastic exposure in *Carassius auratus*. (**a**) Scototaxis index (% time in black and white zones), (**b**) total distance travelled, (**c**) latency to enter the white zone, and (**d**) number of entries into the white zone. Data are presented as mean + SEM (*n* = 12/group). Student’s *t*-test, * *p* < 0.05 compared to the control group. C: control (blue); MP: microplastics (orange).

In the black–white preference test, fish exposed to microplastics showed a significant reduction in the number of entries into the white compartment (Figure 9d). The percentage of time spent in the dark compartment, corresponding to the scototaxis index, tended to increase in the microplastic group (Figure 9a). Moreover, no statistically significant differences were found between groups in total traveled distance (Figure 9b) or latency to first entry (Figure 9c).

### 3.7. Plasma Cortisol Levels

**Figure 10:**
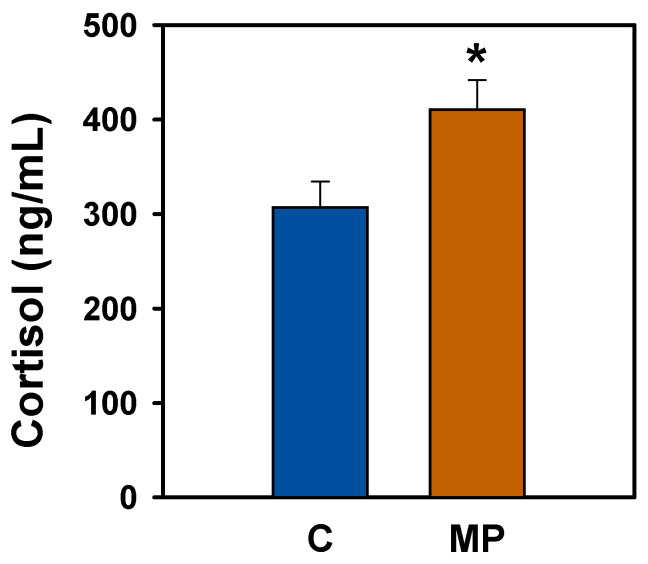
Plasma cortisol levels after microplastic exposure in *Carassius auratus*. Data are presented as mean + SEM (*n* = 11/group). Student’s *t*-test, * *p* < 0.05 compared to the control group. C: control (blue); MP: microplastics (orange).

Fish exposed to microplastics for 14 days showed significantly higher circulating cortisol levels compared to the control group, with an increase of approximately 22% (Figure 10).

## 4. Discussion

This study provides an integrated perspective of the effects of microplastic exposure on energy balance and welfare in fish. By combining behavioral, physiological, metabolic, and neuroendocrine parameters, our results show that microplastics disrupt different components of energy homeostasis, including feed intake, anticipatory feeding behavior, metabolic demand, growth, and stress- and anxiety-related responses. These findings suggest that microplastic exposure induces alterations across central and peripheral regulatory systems, ultimately compromising energy allocation and fish welfare.

### 4.1. Feed Intake and Appetite Regulation

Microplastic exposure significantly reduced feed intake in goldfish, demonstrating a clear anorexigenic effect. This finding agrees with previous observations in the same species [33], with a feeding reduction after a 28-day exposure to microplastics of 50 µm and 50 nm in diameter and is also consistent with reductions observed with larger particles (250–500 µm) in pond loach (*Misgurnus anguillicaudatus*) for 2 weeks of exposure [38]. Our study extends this evidence by showing that the decrease in feeding also occurs with smaller particles (15 µm) and after a considerably shorter exposure (14 days). Notably, average intake was already reduced within the first week, pointing to the fact that brief contact with microplastics is sufficient to elicit a reduction in feeding. This anorexigenic effect was consistently detected using both tank- and individual-level assessments, with an approximate reduction of 15% at the tank level and 45% in individual tests. The larger effect observed in individual measurements likely reflects their higher temporal resolution and more precise endpoints, which enhance the sensitivity for detecting shifts in feed intake compared to tank-level estimates. In addition to reduced consumption, fish exposed to microplastics showed diminished feed anticipatory activity, indicating that these particles interfere not only with consummatory behavior but also with pre-feeding motivation. This pattern is consistent with the effects reported for other anorexigenic pollutants in goldfish, such as the plasticizer DEHP (di-2-ethylhexyl phthalate; [55]).

In fish, reduced feed intake following microplastic exposure has commonly been attributed to the physical consequences of ingesting these particles [6,62,63]. Accumulation in the digestive tract can cause gastrointestinal blockage and stimulate mucus secretion at the intestinal barrier, impairing digestion and nutrient absorption and ultimately evoking a sensation of satiety [23]. Beyond these mechanical effects, however, considerably less attention has been paid to the potential impact of microplastics on neuroendocrine pathways regulating appetite. Our results show marked changes in the expression of key hypothalamic neuropeptides involved in feeding control, with a downregulation of orexigenic peptides transcripts (*npy*, *agrp*, *hcrt*) and an upregulation of anorexigenic ones (*pomca*, *cartpt*). These findings are partially consistent with previous studies. Zhang et al. [33] reported reduced *npy* and increased *crh* (*corticotropin-releasing hormone*) expression in the goldfish hypothalamus, whereas Loganathan et al. [38] observed reduced *hcrt* but no changes in *npy*, *cartpt* or *crh* in pond loach brain. Importantly, despite these species- and particle-size-specific differences, all studies point toward a net anorexigenic balance. In teleosts, the hypothalamus integrates peripheral nutritional signals; among these, leptin plays a central role by inhibiting NPY/AgRP-expressing neurons and stimulating POMC/CART neurons [34,37]. The increase in hepatic *leptin* expression observed in the present study supports the hypothesis that microplastics activate this signaling pathway, leading to a neuroendocrine profile consistent with an anorexigenic state. While the overall transcriptomic balance clearly favors reduced appetite, additional regulatory mechanisms such as post-transcriptional regulation, receptor sensitivity, or potential desensitization processes may contribute to the final feeding response. Notably, this leptin-mediated response may be linked to the concomitant upregulation of *pparα* expression. Activation of PPARα has been associated with anorexigenic effects in goldfish, including increased *leptin* expression and suppression of hypothalamic *npy*, ultimately leading to reduced feed intake [64]. Overall, our results are compatible with the activation of lipid-sensing nuclear receptors by microplastic exposure, leading to changes in peripheral leptin signaling and central appetite-regulating neuropeptides that ultimately modulate feed intake.

In addition to homeostatic control, the reduction in *orexin* expression (*hcrt*) suggests that microplastics may also affect the hedonic component of feeding, since this neuropeptide is involved not only in energy balance but also in reward-related and pleasure aspects of feed intake [65]. Thus, suppression of orexin signaling, also reported by Loganathan et al. [38] in pond loach exposed to microplastics, may contribute to the diminished feed anticipatory activity in exposed goldfish, indicating an impairment of both consummatory and motivational aspects of feeding.

At a peripheral level, an upregulation of *ghrelin* expression was detected in the anterior intestine, the primary site of ghrelin synthesis in goldfish (an agastric species). This finding differs from previous studies in goldfish exposed to microplastics, where reduced circulating ghrelin levels were reported after longer exposure periods [33], whereas no changes in intestinal ghrelin expression were observed in pond loach [38], suggesting that ghrelin responses may depend on the tissue analyzed (intestinal expression vs. plasma levels), exposure duration (14 vs. 28 days), and species-specific differences. Although ghrelin is classically considered an orexigenic signal, its increase in the present study does not translate into elevated feed intake, suggesting a functional dissociation between peripheral ghrelin expression and central feeding regulation under microplastic exposure conditions. Rather than reflecting enhanced orexigenic drive, this increase may represent a compensatory response to reduced feed intake, a typical response to fasting periods observed in this species [66]. Moreover, the role of ghrelin in fish can be context-dependent, since under stress conditions or via interactions with central CRH neurons, it may contribute to anorexigenic outcomes [67,68]. In addition, since microplastics induce intestinal inflammation and mucosal stress [23,69,70], the observed upregulation of intestinal *ghrelin* may also reflect anti-inflammatory and cytoprotective mechanisms aimed at maintaining epithelial integrity [71].

It is also possible that additional peripheral and central signals contribute to microplastic-induced anorexia. In this context, increased intestinal expression of satiety-related peptides such as *cck* and *pyy* [38], activation of stress-related pathways, including increased hypothalamic *crh* expression and elevated plasma levels of ACTH (adrenocorticotropic hormone) and cortisol, as well as higher hypothalamic serotonin levels [33], may further contribute to the anorexigenic effects of microplastic exposure. Taken together, these observations underscore that feeding regulation is a highly integrated and multifactorial process, and that microplastics likely disrupt this balance through the combined disruption of multiple signaling pathways across the gut–brain axis.

### 4.2. Energy Expenditure

Our results show that an increased basal metabolic rate in microplastic-exposed fish is consistent with previous observations in rockfish (*Sebastes schlegelii*; [72]) and grass carp (*Ctenopharyngodon idella*; [73]) after comparable exposure periods (14 days), and even after shorter exposures (4 days) in black sea bass (*Centropristis striata*; [74]). Notably, locomotor activity remained unchanged in our study, indicating that the observed increase in oxygen consumption reflects an elevated metabolic rate and, consequently, higher energetic expenditure, suggesting that microplastic exposure imposes additional metabolic costs that are independent of physical activity. This heightened energy demand likely reflects physiological stress responses, including tissue repair and detoxification processes triggered by microplastics [73,75], as well as oxidative stress and mitochondrial dysfunction. The gills are particularly vulnerable to microplastic exposure due to their direct contact with the surrounding environment, which can lead to particle accumulation and functional impairment [69,76,77,78], potentially contributing to increased oxygen consumption. At the cellular level, microplastics have been shown to impair mitochondrial respiration, disrupt the electron transport chain, and modulate antioxidant defenses [52,78,79,80], effects that may contribute to increased oxygen consumption. This is further supported by evidence in goldfish showing increased oxidative status and antioxidant capacity in gill tissue following nanoplastic exposure [81], highlighting the gills as a main target of nanoparticle-induced stress. Future studies integrating respirometry with enzyme and molecular endpoints —such as antioxidant enzymes like superoxide dismutase, catalase, and glutathione peroxidase, and markers of mitochondrial function— would help to clarify these mechanisms.

Regarding the locomotor activity, no significant changes were detected by microplastic exposure, either in group assessments conducted in the home tanks or in individual open field and black–white preference tests. Previous studies in fish have reported variable locomotor responses, including reduced locomotion [22,25,51,62,72,82] or hyperactivity [83,84,85], highlighting the absence of a uniform locomotor response to microplastics. Differences in species, developmental stage, exposure duration, polymer type, particle size, and concentration have all been suggested as contributing factors to these divergent outcomes. In the present study, the lack of changes in locomotor activity, together with the preservation of daily rhythms in both locomotion and oxygen consumption, suggests that, under our experimental conditions, microplastic exposure does not disrupt general locomotor performance or daily rhythmic patterns associated with energy expenditure. Nevertheless, this does not exclude the possibility that microplastics may affect the circadian system at other levels. Previous studies in zebrafish (*Danio rerio*) have reported that exposure to nanoplastics alters brain physiology, showing phase-dependent differences between light and dark periods in behavioral and molecular responses [86,87]. More recent work has directly shown that microplastic exposure can modify the expression of core circadian clock genes, including *per* and *cry* family members, in the zebrafish brain [25]. Therefore, while daily rhythmic patterns in activity and metabolic rate appear preserved in our experimental setting, targeted chronobiological approaches would be required to determine whether microplastics interfere with the circadian system.

### 4.3. Growth Performance

Several studies support that microplastics generally impair growth across various fish taxa and experimental conditions [8,19,88]. Consistent with this consensus, microplastic exposure in the present study significantly reduced weight gain, length gain, and SGR in goldfish. However, Fulton’s condition factor remained unaffected. This stability in condition factor likely suggests that reductions in weight and length occurred roughly proportionally, likely reflecting the combined effects of reduced feed intake and increased energy expenditure, as described above. These findings suggest that microplastic exposure alters energy allocation and indicate that condition factor may not be sufficiently sensitive to detect sublethal growth impairments over relatively short periods, such as the 14 days of the present study.

In this context, the observed growth impairment appears to be driven by a sustained negative energy balance, where energy intake is insufficient to meet metabolic requirements, leading to the mobilization of endogenous reserves, as reflected by changes in organosomatic indices. Given that the liver is the main energy-storage organ in fish, the decrease observed in the HSI in goldfish suggests a depletion of hepatic energy reserves. Similar reductions in growth and HSI have been reported in *Channa punctata* and are associated with biochemical and histopathological alterations indicative of liver damage [89]. This is in line with evidence that the liver is particularly vulnerable to oxidative stress in fish exposed to microplastics [26,90,91]. A concomitant and slight decrease in perivisceral fat further supports the mobilization of energy reserves under metabolically constrained conditions. In contrast, in marine jacopever (*Sebastes schlegelii*), HSI increased and perivisceral fat remained unchanged despite significant reductions in growth, gross energy, and whole-body protein and lipid content [22,72], indicating that energy allocation strategies under microplastic stress may vary between species.

At the molecular level, these responses are consistent with potential changes in lipid metabolism. The upregulation of *ppara* suggests fatty acid β-oxidation and a greater reliance on endogenous lipid stores to sustain energy demands [92,93]. This metabolic shift is consistent with the observed reduction in hepatosomatic index, indicating depletion of hepatic energy reserves to meet demands under microplastic exposure. PPARα activation is known to promote hepatic lipid catabolism and limit excessive lipid accumulation [94,95], as described for PPARα agonists in fish models. In contrast, the absence of changes in *pparg* expression indicates that lipid storage pathways are not activated, reinforcing a metabolic profile oriented toward energy utilization rather than accumulation [96]. Altogether, these results suggest a stress-induced reprogramming of hepatic energy metabolism characterized by enhanced lipid catabolism and reduced energy storage capacity as part of a compensatory response to microplastic exposure. Future studies should assess key metabolic enzymes and tissue-specific lipid turnover to clarify how these transcriptional responses translate into functional metabolic adjustments.

### 4.4. Anxiety and Stress Responses

Goldfish exposed to microplastics avoided the unprotected areas of the open field and the light side of the black–white preference tank. Importantly, these behavioral changes occurred in the absence of alterations in total distance traveled during the tests or in basal locomotor activity measured over 14 days, indicating that the observed effects are unlikely to be driven by motor impairment, as discussed above. This behavioral profile aligns with an anxiety-like phenotype, as previously described in this species [47]. Comparable anxiogenic effects have been reported in zebrafish exposed to micro- or nanoplastics using different paradigms, including the novel tank, black–white and social preference tests [25,79,97,98]. However, while those studies involved considerably longer exposure periods (21–40 days), the present findings suggest that microplastic-induced anxiogenic effects may emerge after relatively short exposure times (7 days). The reduced exploratory behavior observed in this study is consistent with previous reports in zebrafish [88], crucian carp (*Carassius carassius*; [99]), and marine jacopever [100]. Moreover, microplastic exposure reduced inter-individual distances in zebrafish in shoaling tests, reflecting increased shoal cohesion as a compensatory response to heightened stress or anxiety [25]. Together, these findings suggest that microplastic exposure disrupts multiple behavioral repertoires, including exploration, risk assessment, and social interaction, potentially impairing the ability of fish to appropriately adapt to changing or novel environmental conditions.

Although further studies are needed to determine the molecular processes underlying such behavioral alterations, neurotoxic mechanisms, including increased acetylcholinesterase activity and neuroinflammation, have been suggested [25,101,102]. Our findings suggest that the hypothalamic signaling pathways involved in feeding regulation may also serve as a mechanistic link to anxiety modulation, as neuropeptides involved in energy homeostasis play key roles in anxiety regulation [103,104]. In medaka (*Oryzias latipes*), increased *npy* and *agrp* expression, together with reduced *pomc*, has been associated with anxiolytic effects [105]. In this sense, the hypothalamic neuroendocrine profile observed in our study, characterized by decreased *npy*/*agrp* and increased *pomc*/*cartpt*, aligns with a potential anxiogenic response induced by microplastics. Additionally, peripheral signals may also be involved in this anxiogenic state. Thus, the upregulation of intestinal ghrelin observed here could contribute to this response, as this hormone has recently been implicated in anxiety-related behaviors in goldfish [46]. Together, these data support a complex, multi-level neuroendocrine dysregulation driving the behavioral impacts of microplastic exposure.

In addition, elevated plasma cortisol levels in microplastic-exposed goldfish indicate a clear activation of the stress response, consistent with findings reported in other teleost species [106,107,108,109,110]. This rise in cortisol is most likely mediated by activation of the HPI axis, as supported by previous evidence showing upregulation of hypothalamic *crh* and pituitary *acth* [108], as well as elevated circulating CRH and ACTH levels in microplastic-exposed fish [33]. Such CRH activation may, at least in part, be driven by microplastic-induced neuroinflammatory processes within the hypothalamus, although this remains to be elucidated. Moreover, increased expression of cortisol receptors in metabolically active tissues such as the liver and gills has also been reported [32], suggesting an enhanced tissue sensitivity to cortisol that may further potentiate the physiological effects of HPI axis activation. Stress is known to alter energy allocation and metabolic processes, increasing energetic demands for maintenance and defense, and may limit resources available to somatic growth [111]. In this context, the elevated cortisol levels observed in the present study, together with the increased oxygen consumption, support the existence of a stress-induced rise in metabolic demands in microplastic-exposed goldfish. In parallel, microplastics have been shown to disrupt energy metabolism in fish [112]. Although fasting itself is a physiological stressor that may influence cortisol levels and neuroendocrine responses, both control and exposed fish were subjected to identical fasting conditions, and therefore, this factor is unlikely to explain the differences observed between treatments. Together, these findings suggest that microplastic-induced activation of the stress axis, coupled with increased metabolic expenditure, may contribute to the reduced growth observed in the present study. Nevertheless, reduced feed intake is likely the primary driver of the observed growth reduction, with stress-related metabolic changes playing a secondary role. Further research is needed to clarify the specific effects of microplastics on energy metabolism.

It should be noted that the effects described in this study were induced by exposure to virgin polystyrene microplastics, suggesting that the observed alterations in energy balance as well as anxiety- and stress-related responses could be associated with the particles themselves rather than to plastic additives. This is relevant given that plasticizers such as DEHP have been reported to elicit comparable anorexigenic and behavioral alterations in *Carassius auratus* [55]. In environmentally realistic conditions where microplastics may coexist with plastic additives and other contaminants, combined exposures could potentially contribute to a greater disruption of energy homeostasis and welfare than observed under the present experimental conditions.

## 5. Conclusions

**Figure 11:**
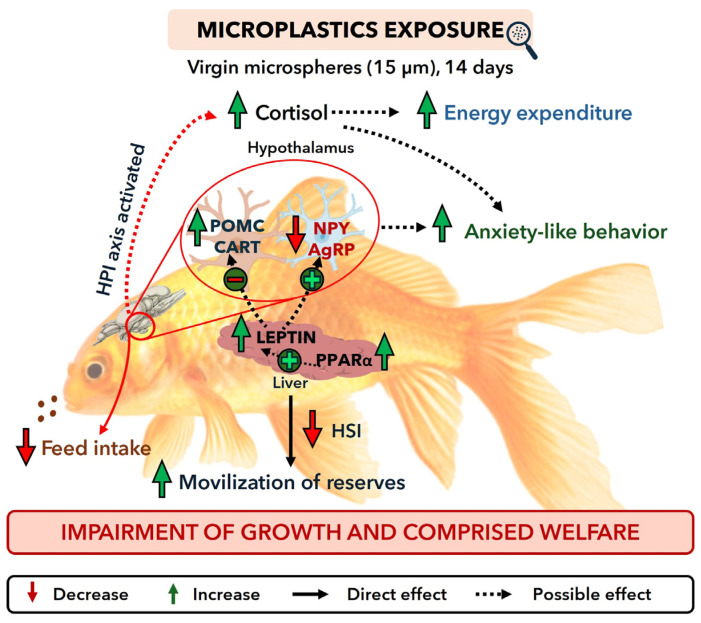
Integrated model of multi-level disruption affecting energy homeostasis and welfare in goldfish exposed to microplastics, highlighting underlying mechanisms. For abbreviations, see the List of Abbreviations.

This study demonstrates that microplastic exposure induces coordinated disruptions in energy balance and emotional state, ultimately compromising overall fish welfare (Figure 11). Exposed fish displayed reduced feed intake and anticipatory feeding activity, together with increased metabolic demands, leading to negative energy balance, constrained somatic growth and depleted hepatic energy reserves. Moreover, the induction of anxiety-like behaviors and activation of the stress axis indicate a clear compromise in welfare. Alterations in appetite-related neuropeptide signaling further suggest that microplastics interfere with shared neuroendocrine pathways regulating anorexigenic and anxiogenic responses. While these findings provide clear evidence under the present experimental conditions, further research is needed to determine their broader applicability across species and environmental contexts. Overall, these findings highlight the high physiological cost of microplastic pollution and emphasize the need for integrated welfare assessments to fully understand its impact on fish welfare and the biological resilience of aquatic organisms.

## Figures and Tables

**Table 1:** Primers access number, sequences and product size for each gene analyzed.

Gen Name	Gen Symbol	Access No. (GenBank)	Sequence (5’ > 3’)	Amplicon (bp)
*beta-actin*	*actb*	LC382464.1	F: CAGGGAGTGATGGTTGGCA R: AACACGCAGCTCATTGTAGA	168
*agouti-related peptide*	*agrp*	AJ555492.1	F: ATGGCATCACATCCAAACC R: GCTTTACCCAGATCCTCATCA	152
*cocaine and amphetamine regulated transcript 1*	*cartpt1*	AY033816.1	F: GTGCCGAGATGGACTTTGAC R: AGCTGCTTCTCGTTGGTCAG	97
*cocaine and amphetamine regulated transcript 2*	*cartpt2*	AY033817.1	F: GGAAAAGCTGCAGACGAAAC R: CGATTCGAGAGCCTTTTCTG	121
*prepo-ghrelin*	*ghrelin*	AF454389.1	F: TTCATGATGAGTGCTCCGTTC R: GTCAGAATTCAAGTGGCGAATC	124
*prepo-orexin, hypocretin*	*hcrt*	DQ923590.1	F: ACTGCACAGCCAAGAGAGTTCA R: GTTATTAAAGCGGCCGATATGC	188
*leptin (ob)*	*lepa1*	FJ534535.1	F: AGCTCCTCATAGGGGATC R: TAGATGTCGTTCTTTCCTTA	192
*neuropeptide Y*	*npy*	M87297.1	F: TTCGTCTGCTTGGGAACTCT R: TGGACCTTTTGCCATACCTC	151
*proopiomelatonocortin a*	*pomca*	AJ431209.1	F: CTCACCACTGACGAGAACATCTTG R: CGGTTTGCTCCAGCTCAGA	121
*peroxisome proliferator-activated receptor alpha*	*ppara*	AY198322.1	F: CCATCCCGACAACGAGTTCC R: CAGCGACGTGTCTTCTGTCT	201
*peroxisome proliferator-activated receptor gamma*	*pparg*	AY894893.1	F: TTCCACAGCTGTCAGTCTCG R: CCTACGGACAGATCTTCAT	121

F: forward, R: reverse.

**Table 2:** Effect of microplastics on growth and organosomatic indices in *Carassius auratus*.

	Control	Microplastics	*p*-Valor
Body Weight Gain (%)	17.27 ± 1.69	13.18 ± 1.4	0.037
Body Length Gain (%)	5.06 ± 0.88	2.95 ± 0.59	0.029
Standard Growth Rate (%/day)	1.22 ± 0.11	0.96 ± 0.1	0.048
Hepatosomatic Index (%)	3.6 ± 0.25	2.98 ± 0.2	0.036
Perivisceral Fat Index (%)	0.54 ± 0.11	0.37 ± 0.03	0.07
Fulton’s condition factor	3.04 ± 0.1	3.14 ± 0.06	0.184
Survival rate (%)	100	100	-

Data is presented as mean ± SEM (*n* = 11–12/group). Student’s *t*-test.

## Data Availability

Data are available in the DOCTA database of UCM (https://hdl.handle.net/20.500.14352/134426 (accessed on 26 April 2026)).

## Supplementary Materials

The following supporting information can be downloaded at: https://www.mdpi.com/article/10.3390/ani16091381/s1, Figure S1: Locomotor activity of *Carassius auratus* in the previous week of the experiment.

## Abbreviations

The following abbreviations are used in this manuscript:

A	Amplitude
*agrp*	Agouti-related peptide
*actb*	Actin beta
ACTH	Adrenocorticotropic hormone
ANOVA	Analysis of variance
BW	Body weight
BWi	Initial body weight
BWf	Final body weight
C	Control group
*cartpt1*	Cocaine and amphetamine regulated transcript 1
*cartpt2*	Cocaine and amphetamine regulated transcript 2
CRH	Corticotropin-releasing hormone
F	Dilution factor
FAA	Feed anticipatory activity
FI	Feed intake
*hcrt*	Hypocretin
HPI	Hypothalamic–pituitary–interrenal
HSI	Hepatosomatic index
*lepa1*	Leptin a1
Li	Initial body length
Lf	Final body length
M	Mesor
MO_2_	Oxygen consumption
MP	Microplastics group
MS-222	Tricaine methanesulfonate
n	Sample size
No	Number
*npy*	Neuropeptide Y
OW	Organ weight
PCR	Polymerase chain reaction
PFI	Perivisceral fat index
*pomca*	Proopiomelatonocortin a
*ppara*	Peroxisome proliferator-activated receptor alpha
*pparg*	Peroxisome proliferator-activated receptor gamma
RNA	Ribonucleic acid
RNasin	RNase inhibitor
RT-qPCR	Real-time quantitative polymerase chain reaction
SEM	Standard error of mean
SNK	Student–Newman–Keuls
SGR	Specific growth rate
Wi	Initial feed weight
Wf	Final feed weight
