# Supplementary material for: Microplastic Exposure Disrupts Energy Homeostasis and Welfare in Goldfish
Source: Animals (Basel). 2026 Apr 30;16(9):1381. doi: 10.3390/ani16091381 (PMC13162591; doi:10.3390/ani16091381)
Supplement: Supplementary file 1 [file animals-16-01381-s001.zip › animals-4250486-supplementary.pdf]

Supplementary material

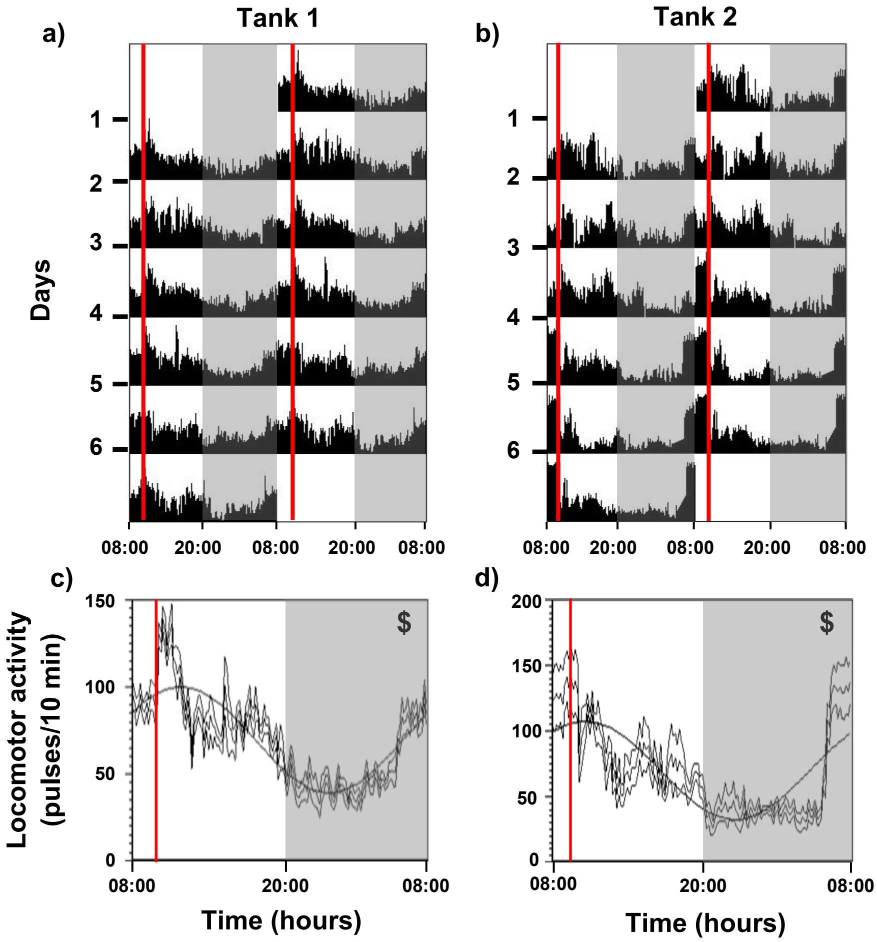

Figure S1. Locomotor activity of *Carassius auratus* the previous week of the experiment. (a-b) Representative actograms for tank 1 (subsequently assigned to the control group) and tank 2 (subsequently assigned to the microplastic group), illustrated in a double plot format (48-h time scale) for better visualization. (c-d) Average waveform of locomotor activity (mean ± SEM, n=6 days), with periodic sinusoidal function wave represented by bold black line. Light and dark phases are indicated by white and grey areas, respectively. The red line shows the feeding time (10:00). Significance of the Rayleigh test for a 24-h rhythm are shown ($ *p < 0.001*).
